# Effect of Optimization of Glycaemic Control on Mannan-Binding Lectin in Type 1 Diabetes

**DOI:** 10.1155/2017/1249729

**Published:** 2017-11-26

**Authors:** Gry Høst Dørflinger, Charlotte Brink Holt, Steffen Thiel, Jakob Appel Østergaard, Troels Krarup Hansen

**Affiliations:** ^1^Department of Endocrinology and Internal Medicine, Aarhus University Hospital, 8000 Aarhus, Denmark; ^2^Department of Biomedicine, Aarhus University, 8000 Aarhus, Denmark

## Abstract

**Objective:**

Mannan-binding lectin (MBL) concentration in plasma is increased in subjects with type 1 diabetes and associated with increased mortality and risk of diabetic nephropathy. Recent findings show that pancreas transplantation reduces MBL concentration. Whether the increased MBL concentration is reversed by improved glycaemic control remains unknown. We investigated the effects of improved glycaemic control on MBL concentration in patients with type 1 diabetes.

**Methods:**

We measured MBL, fructosamine, and HbA_1c_at baseline and after 6 weeks in 52 type 1 diabetic patients following the change from conventional insulin therapy to insulin pump therapy.

**Results:**

After initiation of insulin pump therapy, the total daily insulin dose was significantly reduced (from 51 ± 18 IE/day to 39 ± 13 IE/day, *P* < 0.0001). There was a significant decrease in HbA_1c_ from 8.6% to 7.7% (from 70 mmol/mol to 61 mmol/mol, *P* < 0.0001) and in fructosamine levels (from 356 *μ*mol/L to 311 *μ*mol/L, *P* < 0.0001). MBL levels decreased by 10% from 2165 *μ*g/L (IQR 919–3389 *μ*g/L) at baseline to 1928 *μ*/L (IQR 811–2758 *μ*g/L) at follow-up (*P* = 0.005), but MBL change was not significantly correlated with changes in insulin dose, HbA_1c_, or fructosamine.

**Conclusions:**

MBL concentration decreased following the initiation of insulin pump therapy in patients with type 1 diabetes and did not correlate with changes in glycaemic control.

## 1. Introduction

Diabetic vascular complications progress in part as a consequence of hyperglycaemia. A growing body of evidence links the complement system, in particular mannan-binding lectin (MBL) and the lectin partway, to this pathophysiological process.

MBL is a soluble pattern recognition molecule of the innate immune system that may activate the complement system via the lectin pathway. Concentration of circulating MBL is predominantly determined by the genotype, where polymorphisms give rise to large interindividual differences [1]. Intraindividual differences in MBL concentrations are far smaller [2] and fluctuate as an acute-phase response [3] and from hormonal influence [4, 5].

MBL levels are increased in patients with type 1 diabetes, and high levels of MBL have been associated with increased mortality [6] and development of nephropathy [7–9]. Studies in mice have shown that the absence of MBL minimizes the inflammatory injury induced by ischemia [10–12]. Furthermore, deficiency of MBL protects mice against diabetic nephropathy [13]. On the other hand, low MBL levels have been associated with enhanced risk of myocardial infarction [14] and more pronounced plaque formation [15]. Also, MBL seems to have a beneficial role in the clearance of atherogenic lipoproteins by monocytes and macrophages [16].

MBL levels are increased in type 1 diabetic patients without any association between the genotype and type 1 diabetes [7, 17, 18], but whether this is reversible by improving glycaemic control remains unknown. Animal studies demonstrate a clear association between the increase in blood glucose and an increase in MBL levels following the induction of diabetes [19]. Also, altered MBL concentration was observed following pancreas transplantation in patients with type 1 diabetes [20]. We therefore aimed to investigate the possible association between intensified glycaemic regulation and MBL levels in patients with type 1 diabetes.

## 2. Materials and Methods

We included 52 consecutive patients with type 1 diabetes from our outpatient clinic. The patients were selected for switch from conventional insulin therapy to insulin pump therapy due to poor metabolic control.

Insulin therapy was based on insulin aspart (Novo Nordisk A/S, Bagsværd, Denmark) and administered subcutaneously by a pump system (Minimed 512; Medtronic, Northridge, CA, USA).

Blood samples were collected at baseline and after 6 weeks of treatment with a pump. Serum was isolated from venous blood drawn from the cubital vein and immediately frozen to −80°C until time of analysis.

The study was conducted in accordance with the Helsinki II Declaration and approved by the local Ethics Committee.

### 2.1. Assays

Serum MBL concentrations were measured using an in-house time-resolved immunofluorometric assay with a lower detection level of 10 *μ*g/L [7]. In brief, microtiter wells were coated with mannan followed by incubation with samples diluted 200-fold. After washing, a monoclonal anti-MBL antibody (131-1; Immunolex, Copenhagen, Denmark) labelled with europium using reagents from Wallac Oy (Turku, Finland) was added. After incubation and washing, the amount of the bound europium-labelled antibody was assessed by time-resolved fluorometry (Delphia; Wallac, Turku, Finland). We used two established tests (HbA_1c_ and fructosamine) to monitor the effect of treatment. HbA_1c_ is used to a great extent in our outpatient clinic to monitor glycaemic control; however, fructosamine is only used in special cases as in conditions with changed erythrocyte lifespan (e.g., haemolysis or splenectomy).

Fructosamine concentration was estimated by a commercially available kit (ABX Pentra Fructosamine, Montpellier, France) based on the tetrazolium method. HbA_1c_ was measured at the Clinical Biochemistry Department using gold standard methods.

### 2.2. Statistical Methods

MBL levels were nonnormally distributed, and values were given as medians with interquartile ranges (IQR). All other values were given as means ± SD. To analyse changes in MBL from baseline to follow-up, we used the Wilcoxon signed-rank test, whereas paired Student's *t*-test was used for normally distributed variables. Spearman correlation with two-tailed probability values was used to estimate the strength of association between the observed changes. Statistical significance was assumed at *P* < 0.05. All statistical calculations were performed with IBM SPSS for Windows (version 20; IBM, Armonk, NY, USA).

## 3. Results

**Figure 1:**
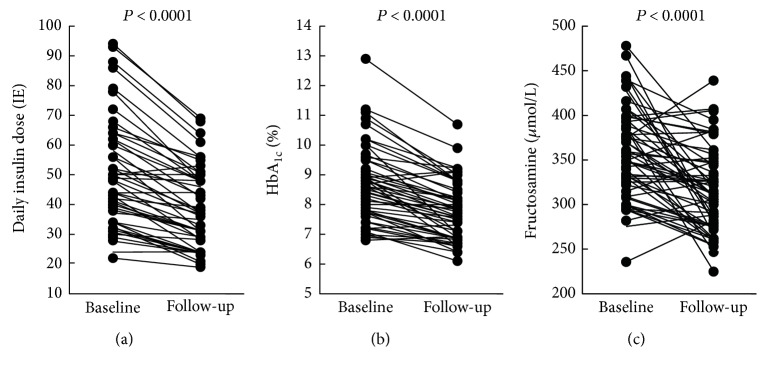
Daily insulin dose (a), HbA_1c_ (b), and plasma levels of fructosamine (c) at baseline and after 6 weeks in 52 type 1 diabetic patients changed from conventional insulin therapy to insulin pump therapy. *P* values refer to the change from baseline to follow-up by Student's *t*-test.

The mean age of the participant was 40 ± 11 years, with average diabetes duration of 21 ± 2 years. After initiation of insulin pump therapy, the patients' total daily insulin dose was significantly reduced (from 51 ± 18 IE/day to 39 ± 13 IE/day, *P* < 0.0001) (Figure 1(a)). Despite this, there was a significant decrease in both HbA_1c_ from 8.6% to 7.7% (from 70 mmol/mol to 61 mmol/mol, *P* < 0.0001) (Figure 1(b)) and fructosamine levels (from 356 *μ*mol/L to 311 *μ*mol/L, *P* < 0.0001) (Figure 1(c)). The relative reductions in HbA_1c_ and fructosamine levels were highly correlated (*ρ* = 0.45, *P* < 0.001) but were not correlated with changes in insulin dose (*ρ* = 0.21, *P* = 0.13 and *ρ* = 0.17, *P* = 0.22, resp.).

**Figure 2:**
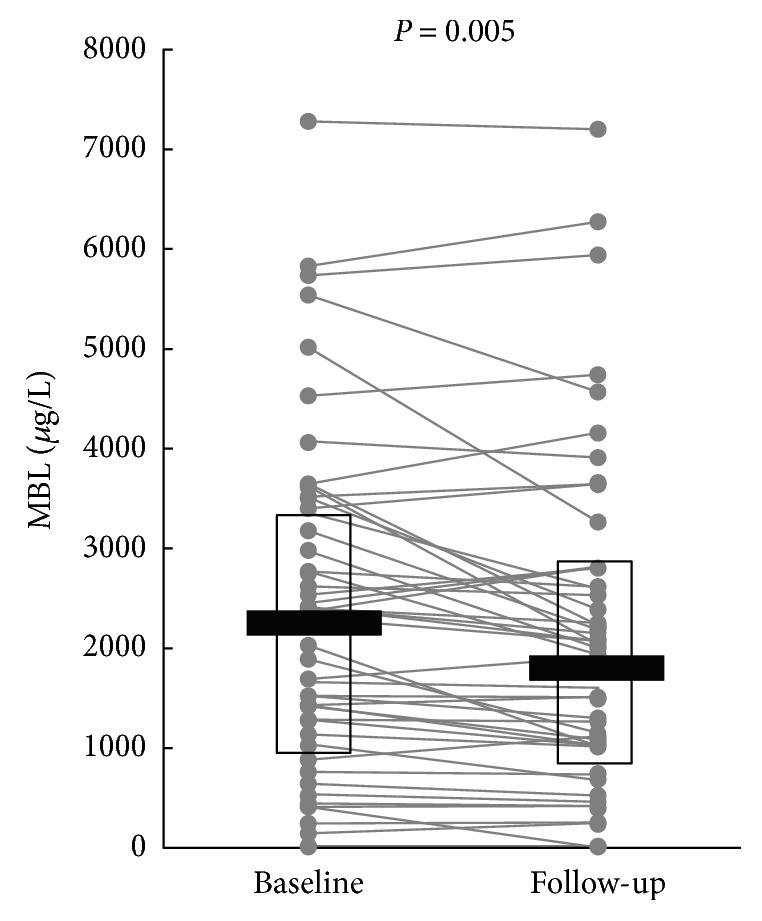
Distribution of serum MBL at baseline and after 6 weeks in 52 type 1 diabetic patients changed from conventional insulin therapy to insulin pump therapy. Horizontal bars represent medians, and boxes indicate IQRs. *P* value refers to the change from baseline to follow-up by the Wilcoxon signed-rank test.

There was a 10% decrease in MBL concentration from 2165 *μ*g/L (IQR 919–3389 *μ*g/L) at baseline to 1928 *μ*g/L (IQR 811–2758 *μ*g/L) at follow-up (*P* = 0.005) (Figure 2). In exploratory analysis, patients were divided into two groups according to the median MBL concentration at baseline (2165 *μ*g/L) as this would indicate either high-expressing MBL genotypes or low-expressing MBL genotypes. In patients with MBL above the median, MBL concentration changed from 3379 *μ*g/L (IQR 2535–4061 *μ*g/L) to 2711 *μ*g/L (IQR 2199–3910 *μ*g/L) (*P* = 0.07) whereas in patients with MBL below the median, MBL concentration changed from 955 *μ*g/L (IQR 399–1429) to 877.5 (252–1149) (*P* = 0.01).

The change in MBL was not significantly correlated with the changes in insulin dose (*ρ* = 0.08, *P* = 0.58), HbA_1c_ (*ρ* = −0.15, *P* = 0.29), or fructosamine (*ρ* = 0.09, *P* = 0.53). Even when the analyses were performed in the subgroup of patients with MBL levels above the median, which would be expected to be carriers of the high-coding MBL genotypes, there were no correlations between changes in MBL and changes in insulin, HbA_1c_, or fructosamine (data is not shown).

## 4. Discussion

Our main goal was to examine the impact of improved glycaemic regulation on MBL levels in patients with type 1 diabetes. We found a significant decline in HbA_1c_ and fructosamine levels in the patients following the change from conventional insulin therapy to insulin pump therapy. In parallel with this, we observed a significant reduction in MBL levels. This was seen despite a significant decrease in total insulin dose used.

It is well established that MBL levels are increased in patients with diabetes compared with healthy control subjects [7, 9, 17, 21]. Animal studies have demonstrated that the rise in MBL is secondary to the induction of diabetes in a streptozotocin model of type 1 diabetes [19]. We originally hypothesized that insulin could have an inhibitory effect on MBL production in the liver. The higher levels of MBL in patients treated with subcutaneous insulin could thus be due to lower concentrations of insulin in the portal system compared to the portal insulin levels seen in subjects with normal pancreatic insulin secretion [17]. We were, however, not able to demonstrate the inhibitory effect of insulin on MBL production from human hepatocytes in *in vitro* studies [22].

It is a common clinical observation that it is possible to obtain significantly lower HbA_1c_ levels with smaller total insulin dose when using insulin pump therapy compared to conventional basal-bolus therapy. In the present study, MBL levels declined despite the use of less insulin, and without any correlation with changes in HbA_1c_ and fructosamine. This seems to indicate that MBL levels are not affected by glycaemic control per se, but rather by other aspects of the type 1 diabetes pathogenesis. Our small sample size may however introduce risk of statistical type 2 errors in our analysis.

Another interesting way of investigating the effect of glycaemic control on circulating levels of MBL is through pancreas-kidney transplantation [20]. The authors observed elevated plasma MBL levels in patients with diabetic nephropathy, which were normalized after pancreas-kidney transplantation. Kidney transplantation in solitude did not have a corresponding effect on MBL levels, indicating that glycaemic control is the driving factor.

In a cross-sectional study, Bouwman et al. [21] found that MBL serum levels as well as MBL complex activity were elevated at diagnosis of type 1 diabetes in juvenile subjects with high MBL-producing genotypes compared to their healthy siblings. For all genotypes, they found MBL complex activity, but not MBL serum levels, to correlate with fructosamine concentrations. The authors hypothesize that the elevated MBL serum level was the result of the immunopathogenesis of type 1 diabetes, whereas the elevated MBL complex activity may be affected by glycaemic control.

We found no correlation between MBL and fructosamine, HbA_1c_, or total insulin dose. A previous study has shown a significant correlation between HbA_1c_ and MBL in patients with type 1 diabetes, especially among patients with high MBL genotypes [7]. MBL genotypes were not available in the present study. We therefore divided patients according to MBL concentration either above or below the MBL median as an indicator of high-expression and low-expression MBL genotypes [17]. However, we still found no correlation between HbA_1c_ and MBL even in the “high MBL concentration” group.

The regulation of MBL production is probably multifactorial, and the immunopathogenesis of diabetes may to some extent take part. Animal studies indicate that both increased production and prolonged half-life of MBL in diabetes may explain the kinetics of the increase in MBL seen in diabetic animals [19]. Despite the absence of a correlation between MBL and HbA_1c_ in our study, it is intriguing to consider hyperglycaemia-induced low-grade inflammation a confounding factor in enhanced MBL production, especially in view of the significant parallel fall in MBL and HbA_1c_ [20]. Furthermore, the relatively short time to follow up of 6 weeks may have disguised a potential correlation. However, using fructosamine to estimate the average level of blood glucose control over a period of 2-3 weeks, we hoped to find a correlation.

A significant decrease in MBL following intensive insulin treatment has previously been described in an intervention study of 451 critically ill patients [3], and in this study, the intensive insulin treatment was also associated with a significant reduction in inflammation as indicated by reduced high-sensitivity C-reactive protein (hs-CRP) levels [3].

In conclusion, MBL levels are significantly reduced following the initiation of insulin pump therapy in type 1 diabetic patients, despite a significant reduction in total insulin dose, but the change was not correlated with indicators of improved glycaemic control.
